# Influence of Cryomilling on Crystallite Size of Aluminum Powder and Spark Plasma Sintered Component

**DOI:** 10.3390/nano12030551

**Published:** 2022-02-06

**Authors:** Amanendra K. Kushwaha, Raven Maccione, Merbin John, Sridhar Lanka, Manoranjan Misra, Pradeep L. Menezes

**Affiliations:** 1 Department of Mechanical Engineering, University of Nevada, Reno, NV 89557, USA; akushwaha@nevada.unr.edu (A.K.K.); rmaccione@nevada.unr.edu (R.M.); merbinjohn@nevada.unr.edu (M.J.); 2 Department of Chemical and Materials Engineering, University of Nevada, Reno, NV 89557, USA; slanka@nevada.unr.edu (S.L.); misra@unr.edu (M.M.)

**Keywords:** nanocrystalline aluminum, cryomilling, spark plasma sintering, synthesis, characterization, microstructures, mechanical properties

## Abstract

The present investigation aims to develop nanocrystalline (NC) pure aluminum powders using cryomilling technique and manufacture bulk components using spark plasma sintering (SPS). The cryomilling was performed on pure Al powders for 2, 6, and 8 h. The cryomilled powders were then consolidated using SPS to produce bulk components. The particle morphology and crystallite size of the powders and the bulk SPS components were analyzed using scanning electron microscopy (SEM), X-ray diffraction (XRD), and transmission electron microscopy (TEM). The results showed that the crystallite size of pure Al powders decreases with increased cryomilling time. The results also showed that the SPS at elevated temperatures resulted in a slight increase in crystallite size, however, the changes were insignificant. The mechanical properties of the bulk components were determined using a Vickers microhardness tester. The hardness of the cryomilled SPS component was determined to be three times higher than that of the unmilled SPS component. The mechanism for the reduction in crystallite size with increasing cryomilling time is discussed. This fundamental study provides an insight into the development of bulk nanomaterials with superior mechanical properties for automotive, aerospace, marine, and nuclear applications.

## 1. Introduction

Over the past decades, conventional polycrystalline materials with coarse grain size have been commonly used for various industrial applications. However, researchers need to find alternative ways when the application demands superior mechanical properties and surface integrity. Severe plastic deformation (SPD) is considered a viable solution for improving surface mechanical properties and surface integrity in polycrystalline materials [1,2,3,4]. These SPD methods can create gradient nanostructured layers on the component’s surface, thereby providing superior fatigue resistance, corrosion resistance, hardness, and other tailored properties. This method can significantly hinder crack initiation and propagation on the component surface and remarkably improve surface mechanical properties. However, SPD techniques can provide enhancement in properties up to a limited depth from the surface. In addition to that, these post-processing techniques are costly. Scholars revealed that adopting nanostructuring can enhance the bulk mechanical properties of the materials and can be effectively used for an application that needs remarkable properties [5].

The concept of nanostructuring to achieve nanocrystalline (NC) materials was first introduced by Birringer et al. [6] in 1984. NC materials are single or multi-phase solids with a mean crystallite size of 100 nm or less [7]. Since then, this concept has been widely accepted and is quite popular among material scientists and engineers. According to the linear Hall–Petch relationship for NC materials, a significant proportion of the crystallites are located on or near the grain boundaries due to the small size of the constituting grains [8]. These act as a barrier for dislocation movement and grain growth, thus creating internal flow stress [9]. As a result of this, NC materials offer superior mechanical properties and are considered as a potential candidate for applications that demand improved properties compared to polycrystalline materials with coarser grain structures [10]. Scholars have reported high hardness [11,12], enhancement in strength [13,14], superior wear resistance [15,16], improved fatigue life [17,18], high thermal expansion coefficient [19,20], and high corrosion resistance [21,22] for NC materials. NC materials are also reported to have seven times higher hardness and ten times higher yield strength as compared to their polycrystalline coarse grain counterparts [23,24]. NC materials possess a unique combination of high strength and ductility, making them ideal for structural and functional applications in diverse sectors, such as automotive, aerospace, marine, and nuclear applications.

NC materials can be produced using different methods, but the commonly employed methods are high-energy ball milling (HEBM) and cryomilling. Among them, cryomilling is a much sought-after energy-efficient and environment-friendly process to synthesize NC materials in bulk quantities. The cryomilling process uses liquid nitrogen (LN2) which is non-toxic to the environment. The cryogenic temperature during the process aids in rapid grain refinement and prevents any hazardous decomposition of materials being cryomilled, ensuring a lesser chance of contamination. The only by-product of the process is nitrogen gas which can be directly released into the air, causing no harm to the environment. The presence of a cryogenic environment also prevents the recovery and recrystallization of refined grains [25]. This ultimately provides a superior crystallite size reduction and significant grain refinement compared to other NC material synthesis methods. Several studies reported the beneficial role of cryomilling on the mechanical properties of various components used for diverse applications. Guan et al. [26] analyzed the effect of cryomilling duration on microstructure evolution and microhardness of AZ31 Mg powders. The authors reported a crystallite size of 26 nm after 6 h of cryomilling, and they revealed that in the initial stages of cryomilling, cold welding of the particles dominated over the fracture, which led to an increase in the crystallite size. However, when the cryomilling time increases, fracture dominates over cold welding, which reduces crystallite size. The authors summarized that the 6 h of cryomilling significantly reduced the crystallite size and provided a remarkable improvement in the hardness of the material. Kumar et al. were able to produce bulk quantities of NC aluminum powder with crystallite size as low as 7 nm to 10 nm [27]. Recently, cryomilling has been actively employed to produce bulk NC powders for various metals and their alloys, thus establishing it as an effective method of manufacturing bulk quantities of NC powders [28,29,30,31,32].

The superior properties of the bulk samples depend upon the preservation of the NC structure during the sample preparation. However, conventional methods of producing bulk samples using powder metallurgy often result in substantial grain coarsening during the consolidation process. Tang et al. [33] experimented with cryomilled Al5083/SiC_p_ composite powders and performed hot isostatic pressing (HIP) to create bulk samples. Their results showed that cryomilled powder with a mean crystallite size of 30 nm undergoes rapid grain growth after HIP followed by hot rolling at 400 °C, with an increase in the crystallite size in the range of 100 nm to 200 nm. In the inter-particle region, the grain size increased to 700 nm, which no longer lies under the NC regime (less than 100 nm). Kishimoto et al. [34] reported an increase in crystallite size in the range of 25 nm to 500 nm during the HIP of 14CrYWTi ferritic alloys. Spark plasma sintering (SPS) is a novel powder metal sintering technique that has been recently employed to prepare nearly dense bulk NC components using metal powders [35,36,37]. SPS is a modified HIP method in which the pulsed-current and the uniaxial pressure are applied at the same time [38]. The heating of the powder sample occurs due to the pulsed current flowing through the die into the sample, which causes rapid heating of the sample at nearly 1000 °C/min [39]. The high temperature allows for the densification of the powdered sample under constant uniaxial pressure. This allows the creation of bulk samples at much lower temperatures and shorter heating cycles than other consolidated techniques. Due to a lower temperature and short heating cycle, the metal powders can retain the NC property, which facilitates the improvement in mechanical properties [40]. The electric discharge generated between the powder particles helps break down the oxide layer on the aluminum particle surface, thus reducing the oxide content in the SPS sample [41]. Although SPS has been used to consolidate different types of metals, ceramics, alloys, etc., the inherent behavior of each of the constituents during the consolidation process affects the properties of the SPS component. In the case of NC materials, the NC grains are susceptible to grain coarsening due to elevated temperatures [42]. Therefore, the sintering parameters such as sintering temperature, uniaxial pressure, and sintering time need to be well established for such unique materials. During the SPS consolidation process, the densification mechanism to produce highly dense components for low melting point metal, such as pure Al with NC grain structure, remains unclear. There is also very limited research on the effect of cryomilled particle morphology, such as an increased surface area for oxidation and uneven surface texture on the SPS sample density and grain growth.

In the current study, the cryomilling process was used to prepare NC pure Al powders, and the SPS process was used to consolidate these powders to achieve a bulk NC sample. The morphology and microstructure evolution of the pure Al powders and the bulk SPS samples were studied using scanning electron microscopy (SEM), X-ray diffraction (XRD), and transmission electron microscopy (TEM). The hardness of the bulk SPS samples was studied using Vickers microhardness testing. The inherent mechanisms of the cryomilling and SPS processes and their effect on the crystallite size and properties of pure Al were analyzed based on the characterization studies.

## 2. Experimental Procedures

### 2.1. Sample Preparation

#### 2.1.1. Powders

**Figure 1:**
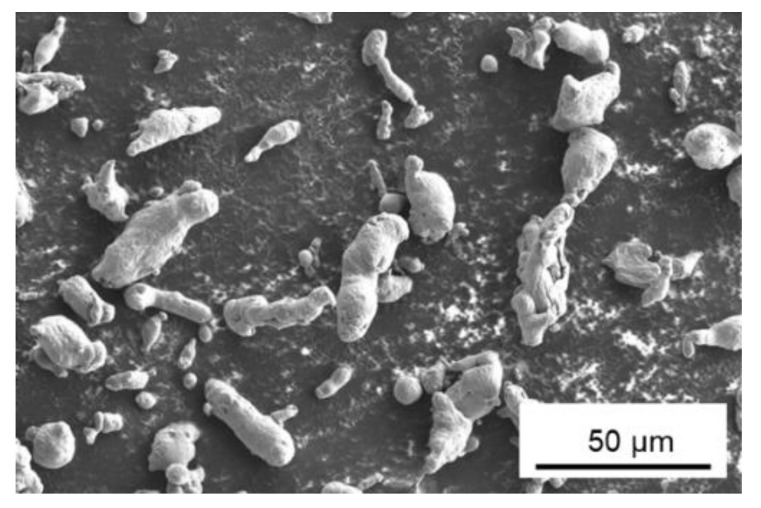
SEM micrograph of as-received Al powders from Alfa Aesar.

To prepare NC powder samples, gas-atomized pure Al powder (product no. 11067) from Alfa Aesar (Haverhill, MA, USA), with −325 mesh and 99.5% purity (trace metals less than 0.5%) was cryomilled for the current study. Pure Al was selected for this study to gain a fundamental understanding of crystallite size reduction using the cryomilling process for Al and its alloys. This study also helps to understand the mechanisms involved in bulk component manufacturing using NC powders at elevated temperatures that can be used to manufacture other NC alloys with enhanced mechanical properties. Figure 1 shows the morphology of as-received Al powder particles which have an irregular cylindrical shape. The powders were stored in airtight containers under a nitrogen environment in a glove box to prevent oxidation, with silica packets to remove any water from the ambient environment before and after cryomilling.

**Figure 2:**
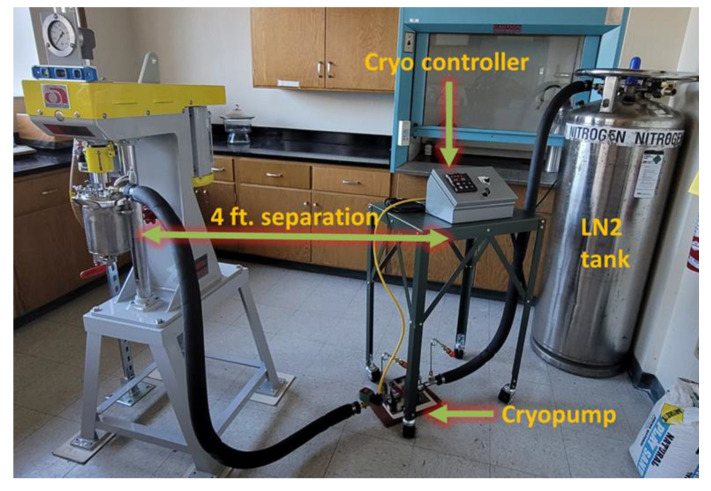
Cryomilling setup at the University of Nevada, Reno.

The cryomilling of the powders was performed using a modified Union Process 01-ST attritor mill. To prepare the setup for cryomilling, the attrition chamber was moved into the glove box under a nitrogen environment to avoid the exposure of Al powder to atmospheric oxygen, thus preventing oxidation. First, a layer of stainless steel (SS) milling media of 6 mm diameter was added to the attrition chamber to cover the bottom surface. The Al powder was then evenly spread on top of this milling media layer. 0.1 wt.% of stearic acid (C_18_H_36_O_2_) was also added per 100 g of pure Al powder as a process control agent to avoid the agglomeration of the particles during the cryomilling process. Followed by the addition of powders, the remaining milling media was then added to sandwich all the powders in between the milling media layers. Finally, the milling rod was inserted into the chamber and the lid was secured at the top of the chamber, including the seal and lubricant that hold the milling rod in place. This loaded and sealed chamber was then removed from the glovebox and attached to the milling frame. The chamber was hooked up to a liquid nitrogen tank pumping directly into the attrition chamber to ensure the powder remains at cryogenic temperatures and to displace any oxygen. The exhaust from the chamber was hooked up to a fume hood to minimize the exhaust metal powder dispersion into the room. Figure 2 shows the cryomilling setup used for the experiment at the University of Nevada, Reno, NV, USA [5].

The Al powder was cryomilled with a ball to powder ratio of 30:1 at 180 rpm for 2, 6, and 8 h. The nitrogen was supplied continuously to the attrition chamber through a cryo-pump using a cryo-controller, at a constant pressure of 16 psi which maintains the chamber temperature at around −190 °C. The temperature was monitored continuously for the entire duration of the experiment. After completion of the cryomilling process, the chamber was allowed to warm up between −10 °C to 4 °C for the extraction process. The chamber was then removed from the milling frame and wiped clean before being placed back in the glovebox to remove excess moisture from condensation. The glovebox was then sealed and again filled with nitrogen for the extraction process. The powder and media were poured onto a sieve to separate the powder from the media. The milled powder was then collected and packed in an airtight container for material characterization and preparation of bulk SPS samples.

#### 2.1.2. Bulk SPS Consolidated Samples

**Figure 3:**
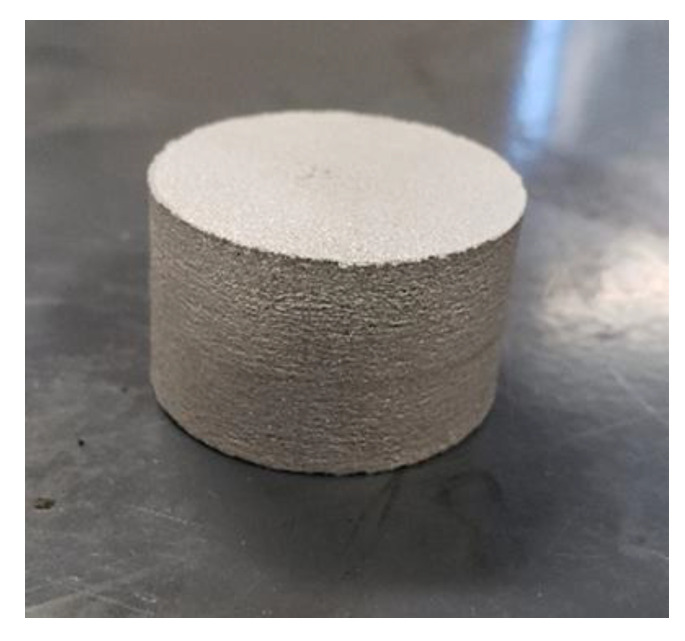
8 h cryomilled pure Al bulk sample manufactured by SPS process.

The bulk SPS components were prepared with unmilled and cryomilled pure Al powders at California Nanotechnologies (Cerritos, CA, USA) using a 211XL SPS machine. 20-g powders samples were formed at 500 °C under 100 MPa pressure using a 25.4 mm diameter mold. Preliminary tests were conducted to determine the ideal time of consolidation for each type of sample. The raw/unmilled pure Al samples were sintered for around 30 min whereas the 8 h cryomilled samples were sintered for around 15 min. The prepared SPS samples were 12.7 mm tall and 25.4 mm in diameter, as shown in Figure 3. The density of the SPS samples was determined using the Archimedes principle. While the SPS samples for raw/unmilled powders had a density value of 99.3% of theoretical density (TD), the samples prepared with cryomilled powders had a density of 98% of TD. The small difference in the density arises due to the varying morphology of the unmilled and milled powders. During the cryomilling process, the repeated welding and fracturing of the particles leads to the formation of crevices and voids on the surface of the particles. This uneven texture of the particle surface traps air between multiple particles during the SPS process causing the formation of microscopic voids. As a result, the density of milled particles is slightly lower than the unmilled particles.

### 2.2. Characterization

#### 2.2.1. X-ray Diffraction Analysis

The unmilled and cryomilled pure Al powders were analyzed using Bruker D8 advance X-ray diffractometer (Billerica, MA, USA) by Cu K_α_ (*λ* = 0.154178 nm) radiation to determine the phase composition and the crystallite size. Five XRD diffraction peaks were used to determine the phases using the JCPDS data for Pure Al. The crystallite size was calculated by the measurement of the peak broadening method using a linear fitting to obtain the Full Width at Half Maximum (FWHM) [43]. The Williamson–Hall method was used to compute the crystallite size of the nanomaterials by determining the total broadening of the peaks [44]. The total broadening ($\beta_{T}$) is equal to the sum of broadening due to Strain $\left(\beta_{\varepsilon }\right)$ and the broadening due to crystallite size $\left(\beta_{D}\right)$. The total broadening is given by the following Equation (1):

(1) $$\beta_{T}\cos\theta =\varepsilon \;\left(4\sin\theta \right)+\frac{K\lambda }{D}\;$$

**Figure 4:**
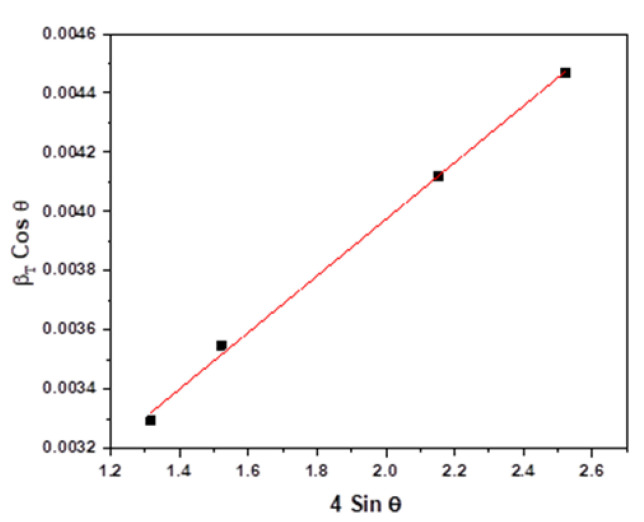
Straight line fit for unmilled Al powder based on the Williamson–Hall equation.

Here, ε is the strain, *θ* is the Bragg’s angle, K is the Scherrer’s constant, *λ* is the wavelength of X-ray, and *D* is the crystallite size. The above equation represents a straight line when $\beta_{T}\cos\theta$ is plotted against 4sinθ. The slope of the straight line is ε and y-intercept is $\frac{K\lambda }{D}$. A straight-line curve fitting of this equation, as shown in Figure 4, indicates that the Al powder is completely random and has no preferential orientation (texture). This straight-line fit has a high value of correlation coefficient (*r*^2^) which indicates that the crystallite size can be accurately calculated using this method. However, for cryomilled powders, there is a deviation from the straight-line fit reducing the value of *r*^2^ which decreases the accuracy of the crystallite size measurement. With the increase in cryomilling time, the net contribution in the broadening of the XRD peaks from the strain energy increases as compared to the broadening due to the reduction in crystallite size [45]. Hence, for higher cryomilling durations, the Williamson–Hall analysis cannot be used to compute the crystallite size accurately.

In general, the peaks in an XRD pattern start to broaden with the increase in milling time, resulting in the decrement of crystallite size. This broadening of peaks can be measured at the FWHM to determine the crystallite size using the Scherrer’s Equation (2) as given below:

(2) $$L=\frac{K\lambda }{B\left(2\theta \right)\cos\theta }\;$$

Here, *B*(2*θ*) is the FWHM of an individual XRD Peak, *θ* is the Bragg’s angle, *λ* is the wavelength of X-ray, *L* is the determined crystallite size, and K is the Scherrer’s constant. The value of K is 0.94 for pure aluminum as it has a cubic symmetry.

#### 2.2.2. Transmission Electron Microscopy Analysis

Analysis of the crystal structure, grain size, and the chemical composition within the microstructure of pure Al and cryomilled powder particles were also performed utilizing TEM (JEOL 2800 STEM field-emission gun, Tokyo, Japan) at 200 keV coupled with dual energy dispersive X-ray spectroscopy (EDS) detectors. The elemental and chemical composition of the pure Al powder particles and bulk SPS samples were determined using these dual EDS detectors.

The Al powder samples were prepared for TEM by dispersing the dry powders onto the TEM grid: ultrathin carbon type A, 400 mesh copper (TedPella, Redding, CA, USA, Product no. 01822). For SPS samples, thin metal shavings were milled out and a focused ion beam (FIB) lamella was prepared using a standard procedure on a ThermoFisher (Waltham, MA, USA) FEI Helios Nanolab 650 equipment [46]. The region of interest was located using the SEM. An electron beam deposited platinum protective layer was deposited with approximate dimensions 10 μm × 1 μm × 1 μm. A second protective layer was then deposited using an ion beam deposited platinum. Trenches were milled on both sides of the protective layer. A micromanipulator was brought near to the surface and attached with a platinum weld. The sides were cut free, and the lift-out section was then moved to a support grid. Once there, it was attached to another platinum weld and then the manipulator was cut free. The lift-out was then thinned out using a series of decreasing ion beam voltages: 30 kV, 16 kV, 8 kV, and a final polishing using 2 kV.

The HRTEM images of the nanoparticles were analyzed using ImageJ software to obtain the d-spacing from the crystalline structure. The fringe pattern in the HRTEM image shows the individual atomic layers that can be measured by directly drawing a selection line. This selection line results in a profile plot that can be measured across the maximum points to determine the d-spacing. However, the image contrast may result in a poor profile plot, in turn affecting the accuracy of d-spacing determination. In the current study, the fringe pattern is first converted into Fast Fourier Transform (FFT) followed by inverse FFT after selecting specific bright diffraction spots in the FFT image. This provides a clear image with high contrast which can be analyzed by plotting the profile by a selection line drawn across the fringe pattern to determine the d-spacing. This d-spacing is used to determine the crystallographic plane for the bright spots by referring to the corresponding JCPDS card for Pure Al. The d-spacing can be calculated from the plotted profile using the following Equation (3):

(3) $$d\text{-}\mathrm{spacing}\;=\;\frac{Total\;length\;of\;plot\;profile\;in\;nm}{Total\;number\;of\;profile\;peaks\;in\;profile\;length}\;$$

#### 2.2.3. Scanning Electron Microscopy Analysis

The particle morphology and microstructure of the pure Al and cryomilled Al powders were examined by field-emission SEM (FEI Quanta 600 FEG, Waltham, MA, USA). The SEM images were taken at 10 kV at multiple magnification levels up to 5000×. Elemental analysis of cryomilled powder particle surface was also performed in SEM using EDS by measuring the energy and intensity of X-rays generated by the electron beam on the sample.

#### 2.2.4. Study the Effect of Annealing Temperature on Grain Coarsening

Due to the small crystallite size and large grain boundary percentage, the crystallites are susceptible to grain coarsening behavior at elevated temperatures due to annealing. To understand this grain coarsening behavior of cryomilled powders, samples were subjected to annealing temperatures in a Thermo Scientific (Waltham, MA, USA) Lindberg/Blue M LGO 1200 °C BF 51842 Series Box Furnace for 1 h at 300 °C, 400 °C, 500 °C, and 550 °C, and allowed to air cool. Post-annealing, the crystallite sizes were determined using XRD to understand the effect of varied annealing temperatures on the grain coarsening.

#### 2.2.5. Microhardness Testing

The microhardness testing was performed on a Tukon 1202 (Buehler, Lake Bluff, IL, USA) microhardness testing machine using a Vickers diamond indenter tip at an indentation load of 50 g with a total indentation time of 15 s, and a dwell time of 10 s. To prepare the samples for microhardness testing, each SPS sample was cut using a diamond disk precision cutter and cold mounted using an epoxy resin-hardener in a 31.75 mm diameter mold. The top surface of the mounted samples was then polished with up to a 1 µm diamond polish slurry to get a mirror finish. For microhardness testing, the samples were secured to the indentation platform by securing tabs that ran parallel to the platform. Ten or more indents were performed on the polished top surface of the SPS sample across the surface. Any exceptionally low hardness measurement values were considered erroneous. These lower values were most likely measured when the indenter was on top of or in close vicinity of a porosity. These values were ignored during the microhardness calculations.

## 3. Results and Discussion

### 3.1. Cryomilled Powders

**Figure 5:**
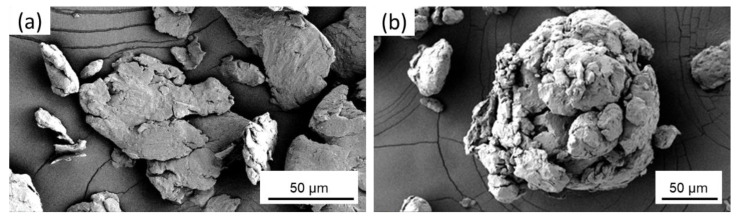
SEM micrographs of pure Al powders after (**a**) 2 h, and (**b**) 6 h of cryomilling.

**Figure 6:**
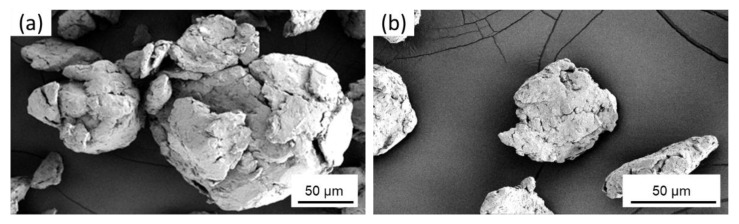
SEM micrographs of pure Al powders after 8 h of cryomilling as seen (**a**) agglomerating to form large particles, and (**b**) broken apart into small particles.

Cryomilling of the powders was performed in the mechanical attritor at cryogenic temperatures. During the cryomilling process, the Al particles undergo repeated fracturing, shearing, cold welding, and re-fracturing under the forces of SS milling media impacting with each other [47]. Due to this repeated fracturing and cold welding of the particles, the aspherical particles become flattened after 2 h of cryomilling as seen in Figure 5a. On further cryomilling, these particles start agglomerating together to form spherical structures that are much larger than the original powder, as seen after 6 h of cryomilling in Figure 5b. The surface of these agglomerated particles starts to smoothen out on further cryomilling. An individual agglomerated particle with flattened surface texture after 8 h of cryomilling is shown in Figure 6a. These bigger particles also break apart into smaller particles of less than 50 µm, as seen in Figure 6b. It can also be observed from the SEM images that the milled Al particles, after 8 h of cryomilling, have a lot of fractures and pores.

**Figure 7:**
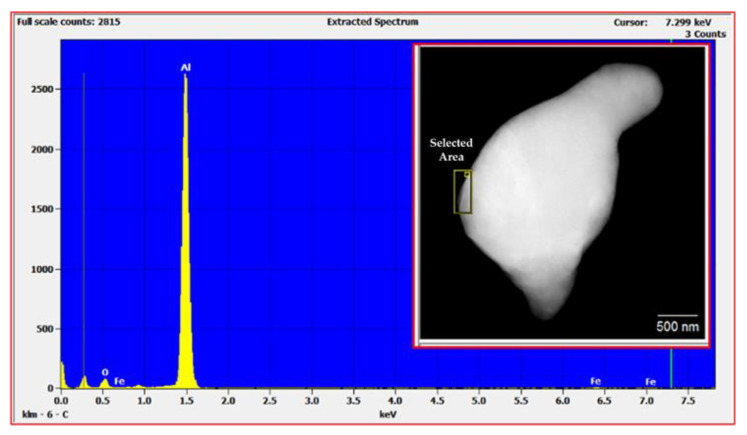
EDS plot for the unmilled powder particle surface showing elemental peaks for Al and oxygen and the corresponding particle (inset image) as determined using TEM.

**Figure 8:**
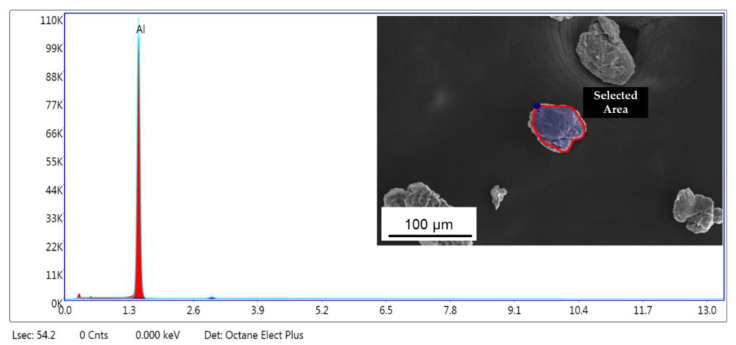
EDS plot for the 8 h cryomilled powders showing elemental peak for Al and the corresponding particle (inset image) as determined using SEM.

During the processing of cryomilled powders, they are mostly maintained in a nitrogen environment to prevent oxidation. However, to understand the oxidation impact on the as-received powders, EDS studies were conducted on the unmilled particle surface. The EDS elemental analysis in TEM for unmilled pure Al particle surface showed the presence of 5.82% oxygen on the surface of the particle, indicating surface oxidation of as-received pure Al. This occurs due to short periods of exposure of metal powders to atmospheric oxygen since pure Al is highly susceptible to oxidation. Figure 7 shows the EDS plot for the unmilled powder particle surface, showing elemental peaks for Al and oxygen. The inset of Figure 7 shows the corresponding unmilled pure Al particle as determined using TEM. The selected area shown on the surface of the particle in the inset figure was characterized as part of particle surface elemental analysis. During cryomilling of this as received pure Al powders, the oxidized surface on the unmilled particles gets mixed and agglomerated inside the particle body due to the repeated fracturing and welding. This agglomerated oxygen might have an impact on the particle properties. Thus, EDS studies were also conducted using SEM on cryomilled powder particles showing that the particles were composed of 99% pure Al, indicating no contamination during the cryomilling process. It also indicated that oxygen is present in trace amounts which will not have any impact on the particle property. Figure 8 shows the EDS plot for the 8 h cryomilled sample particle, showing peak for pure Al only. The inset of Figure 8 shows the corresponding 8 h cryomilled particle (selected area) that was subjected to EDS characterization.

**Figure 9:**
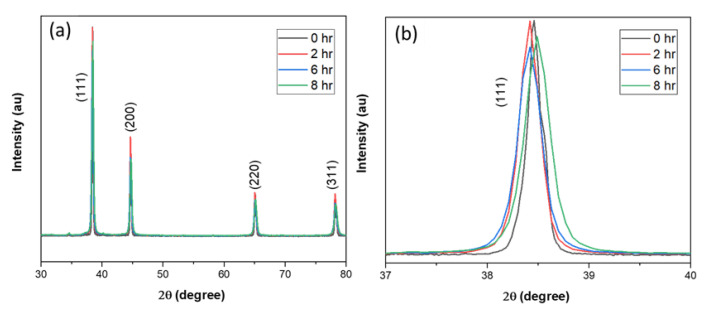
XRD pattern for (**a**) 0, 2, 6, and 8 h cryomilled pure Al powders (**b**) (111) plane peaks for cryomilled pure Al.

The XRD pattern of the cryomilled powder particles showed the material phase composed of pure Al with no impurities (JCPDS Card No. 03-065-2869). XRD peak intensity matching to the JCPDS card indicates that the Al powder is completely random and has no preferential orientation (texture) [30]. Figure 9a shows the XRD patterns for 0, 2, 6, and 8 h cryomilled Al powders. Sharp XRD peaks for all four powder samples indicate that the Al powder is completely crystalline before and after the cryomilling process. Figure 9b shows the broadening and shifting of the characteristic (111) peaks for unmilled and 2, 6, and 8 h cryomilled pure Al powders. The peak shift however does not indicate any specific pattern and thus was assumed to be a device error. For the XRD peak broadening, it is clear that for unmilled pure Al powders, the peak is much narrower than that of the 8 h cryomilled powders. It is also observed that with the increase in cryomilling time the corresponding XRD peaks tend to broaden, which indicates the reduction in crystallite size and increase in strain energy as suggested by the Williamson–Hall equation. From the Williamson–Hall analysis, the correlation coefficient of the linear fit (*r*^2^) for the unmilled pure Al was determined to be 0.99 and for the 2 h cryomilled sample, it was 0.98. This value further reduces to 0.94 and 0.92 for 6 h and 8 h cryomilled sample, respectively, causing a deviation from the straight-line fit. This deviation results in decreased accuracy for the crystallite size measurement [44,45]. Scherrer’s equation was used to overcome this limitation for the crystallite size determination. The broadening of the XRD peak results in increasing the FWHM, which has an inverse relationship with the crystallite size in Scherrer’s equation. The value of K for pure Al is 0.94 and the wavelength (λ) is 1.541178 Å for the Cu radiation tube.

**Figure 10:**
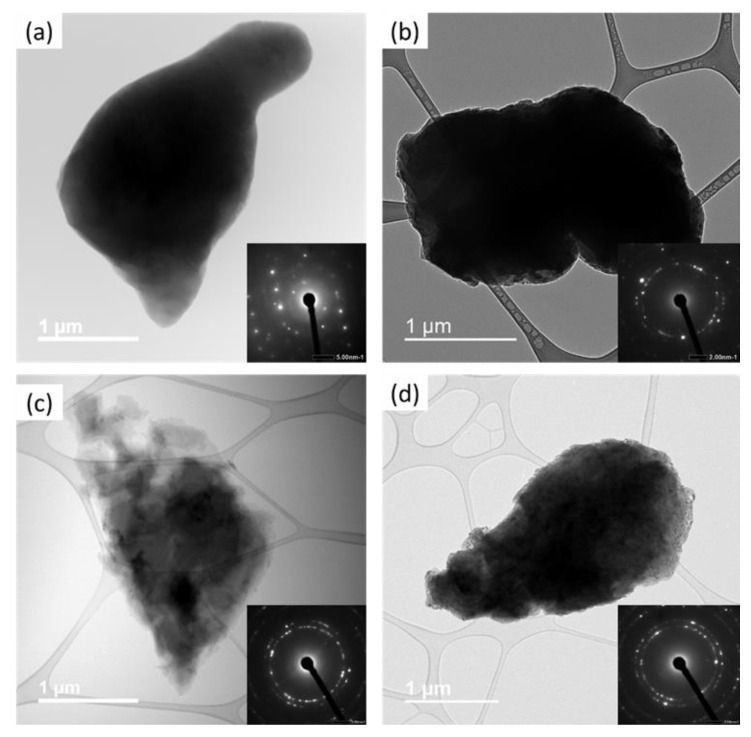
TEM results showing bright-field images and SAD pattern (inset image) for (**a**) unmilled (**b**) 2 h, (**c**) 6 h, and (**d**) 8 h cryomilled samples.

The nanostructuring and the polycrystallinity of the cryomilled powders were verified with the bright-field TEM image. Figure 10a–d shows the bright-field TEM images for the unmilled, 2, 6, and 8 h cryomilled pure Al powder particles. The inset of each of these bright-field TEM images shows the associated selected area diffraction (SAD) pattern having sharp spots and concentric ring pattern, conclusively indicating the polycrystalline nature of the material. SAD patterning was used to determine the crystal structure, lattice parameters, and crystallinity of the material. The SAD pattern was formed by focusing an electron beam through the sample and recording the diffracted beams from different crystallographic planes diffracting at certain angles. The diffracted beams were projected onto the phosphor screen, which leave corresponding bright spots. In this SAD pattern, each point represents a single crystallographic plane direction. These sharp spots in the SAD pattern can be analyzed to determine the specific plane associated with the corresponding spots. In the inset of Figure 10a, the SAD pattern for unmilled pure Al shows a diffraction pattern closer to that of a single crystal with a few distinct bright spots at regular spacing and a specific pattern. However, the appearance of a few non-regular bright spots indicates diffraction from more than one crystallite in the diffraction area. Thus, the powder particles have a polycrystalline nature with a larger crystallite size. In the inset of Figure 10b–d, the SAD pattern for the 2, 6, and 8 h cryomilled samples, the SAD pattern shows a ring of bright spots, which indicates the diffraction from many crystallites in the diffraction area. Thus, it can be concluded that the crystallites are much smaller for cryomilled powders, to accommodate many more crystallites in a smaller diffraction area. The small size of the crystallites is also indicated by the reduced brightness in the outer rings, as the crystallographic planes for these rings have larger atomic spacing and show the existence of NC crystallite structures. A few less bright spots in the SAD pattern are due to the existence of finer crystallites (up to a few 100 nm) in the same diffraction area.

**Figure 11:**
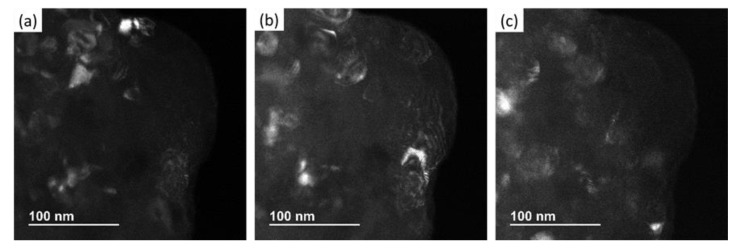
TEM results showing dark-field images of the (**a**) (111) plane (**b**) (200) plane, and (**c**) (220) plane in SAD pattern of 8 h cryomilled pure Al powders.

Dark-field imaging was used to determine the portion of the image that contributed to a particular bright spot in the SAD pattern. These spots were measured for each of the dark field images, using ImageJ software to determine the crystallite size of the material. Figure 11a–c shows the dark field TEM images for the (111), (200), and (220) crystallographic planes for 8 h cryomilled sample. The crystallite sizes as measured using the dark field TEM image analysis are 18 nm for (111) plane, 22 nm for (200) plane, and 16 nm for (220) plane. Thus, the average crystallite size as determined by the SAD pattern in TEM is 19 nm, which complies with the XRD measurement. Table 1 shows the comparison of calculated crystallite sizes for the unmilled, 2, 6, and 8 h cryomilled powders. The XRD results show a 35% decrease in the crystallite size for 8 h cryomilled powder as compared to unmilled powder. The crystallite size for unmilled pure Al was determined to be 43 nm, which was reduced to 28 nm after 8 h of cryomilling. Similar results were observed in TEM studies as well, where the crystallite size is 67 nm for unmilled pure Al which reduces to 19 nm after 8 h of cryomilling. This reduction in crystallite size is about 72%. Thus, it is evident from both XRD and TEM studies that the average crystallite size of the pure Al reduces with the increase in milling time.

**Figure 12:**
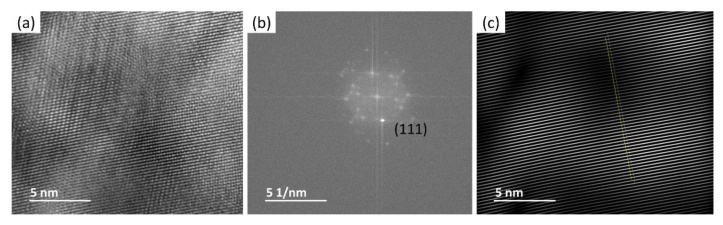
HRTEM results showing (**a**) fringe pattern for the cropped image, (**b**) FFT processed image, and (**c**) an inverse FFT processed image with an inscribed selection line for 8 h cryomilled powder particle.

To determine the specific crystallographic planes, the HRTEM images were analyzed using ImageJ software to obtain the d-spacing from the crystalline structure. Figure 12a shows the HRTEM fringe pattern for 8 h cryomilled sample. This image was processed using the ImageJ software to obtain a FFT image as shown in Figure 12b. Here, all the bright spots corresponding to crystallographic planes are visible. The image was then converted to an inverse FFT image after selecting a few bright diffraction spots. Figure 12c shows the high contrast inverse FFT image with distinct fringe pattern lines along with a drawn selection line running perpendicular to the fringe pattern. The selection line is drawn to generate the plot profile for the fringe pattern.

**Figure 13:**
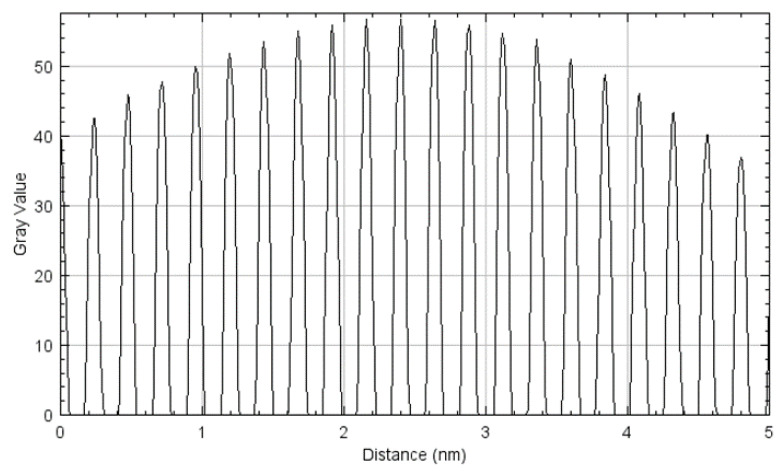
Plotted profile generated in the ImageJ software for the inverse FFT image obtained from the HRTEM image of 8 h cryomilled powder particle.

Figure 13 shows the plotted profile of the fringe pattern generated in the ImageJ software using the selection line drawn in Figure 12c. This plot profile can be measured up to the maximum distance to calculate the d-spacing. This d-spacing value is used to determine the crystallographic plane for the bright spots by referring to the corresponding JCPDS Card for Pure Al. The d-spacing was calculated from Figure 13 using Equation (3).

$$d\text{-}\mathrm{spacing}=\frac{5\;\mathrm{nm}\;}{21\;fringes\;}=0.2381\;\mathrm{nm}$$

#### Effect of Annealing Temperatures on Grain Coarsening of Cryomilled Powders

The production of bulk samples was carried out using SPS consolidation at elevated temperatures. Since the cryomilled powders have a very fine crystallite size, they are susceptible to strain relaxation, grain growth, and recrystallization at elevated temperatures [48]. Thus, the SPS process may cause grain coarsening of the cryomilled pure Al, which may ruin the nanocrystalline aspect of the manufactured bulk sample. To understand the effect of different annealing temperatures on the crystallite size, the cryomilled powders were subjected to elevated temperatures of 300 °C, 400 °C, 500 °C, and 550 °C for 1 h and allowed to cool in air. The crystallite size of the annealed samples was determined using XRD. Table 2 shows the crystallite size measurement for 8 h cryomilled pure Al powder sample, which was annealed at different temperatures. It is evident from the table that the crystallite size increases with the increase in annealing temperatures. This increase in crystallite size is attributed to the grain coarsening and strain relaxation at elevated temperatures. The crystallite size, as determined for 8 h cryomilled sample annealed at 300 °C, is 27 nm, and for the sample annealed at 550 °C, the crystallite size is 34 nm. Although there is a slight increase in the crystallite size, this change is insignificant because the crystallite sizes of annealed samples are still well in the NC range, which will provide the desired improvement in the mechanical properties of SPS samples. This test ensured that the samples could withstand short heat treatment cycles without undergoing considerable change in the crystallite size.

### 3.2. Bulk SPS Consolidated Samples

The unmilled and 2, 6, and 8 h cryomilled Al powders were consolidated using SPS process to produce 12.7 mm tall and 25.4 mm diameter bulk SPS samples with density of 98% of TD. The XRD patterns for SPS samples exhibit sharp peaks, indicating that the pure Al has retained its crystalline structure even after the SPS process. Table 3 shows the XRD crystallite size calculation for unmilled, 2, and 8 h cryomilled and SPS samples as determined from Scherrer’s equation. It is also observed that the average crystallite size for SPS samples increased when compared to the as-cryomilled powders. The average crystallite size for the SPS sample increased by around 46% from 43 nm for unmilled powders to 63 nm after SPS. For 2 h cryomilled sample, the average crystallite size increased by nearly 70% from 35 nm for milled powders to 60 nm after SPS. Similarly, for 8 h cryomilled sample, the crystallite size showed an increment of 55% from 28 nm for powders to 43 nm after SPS. This increase in crystallite size is attributed to the grain coarsening, strain relaxation, and recrystallization that occurs at elevated temperatures (500 °C) during the SPS process. Still, the crystallite sizes of the bulk samples lie in the nanocrystalline range. Another likely reason for this restrained grain growth may be due to the rapid heating cycle, the applied pressure, and the pinning effect of the aluminum oxide on the surface during the consolidation process [49,50].

**Figure 14:**
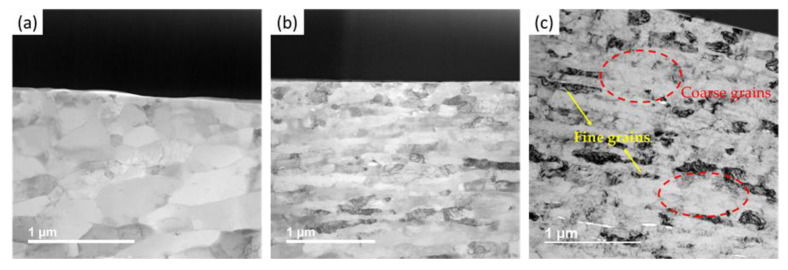
Dark-field TEM images for (**a**) unmilled pure Al SPS sample (**b**) 8 h cryomilled SPS sample (**c**) 8 h cryomilled SPS sample showing regions of fine and coarse grain structure.

To understand the nanostructure of the pure Al SPS sample, bright field TEM studies were conducted on the thin sections of SPS samples. These thin samples were prepared using FIB standard procedures to create ultra-thin lamella suitable for TEM [51,52]. The samples were prepared for unmilled and 8 h cryomilled samples to understand the effect of cryomilling and SPS on the nanostructure of pure Al. Figure 14a shows the dark-field TEM image for the unmilled pure Al SPS sample, showing closely packed equiaxed coarse grain structures with average widths varying from 38 nm to 400 nm in size. This structure is similar to the recrystallization structure of pure Al, manufactured by other methods [53]. However, for the 8 h cryomilled SPS sample, the grain morphology was a bit different. Figure 14b shows a band-like structure with elongated grains for 8 h cryomilled SPS samples where the grain sizes are much finer than that of the unmilled sample. These elongated grains are not equiaxed and stretched out in a particular direction corresponding to all the adjacent grains. These elongated grains have an average width varying from 30 nm to around 200 nm. In comparison to the unmilled sample, this reduction in grain size is attributed to the cryomilling process. High strain rates during the cryomilling process are responsible for the elongation of the grain structure. Severe plastic deformation, diffusion, and viscous flow during the SPS process are responsible for the further densification of the grain structure, which is observed for both the unmilled and milled SPS samples. Figure 14c shows a similar region of interest from the bright-field TEM image of the 8 h cryomilled SPS sample. It shows specific regions of coarser grains within the bulk sample. These irregular-shaped coarse grain structures are much larger than the adjacent finer grain structures. These regions may have formed due to abnormal grain coarsening during the elevated temperatures during the SPS consolidation or may be due to the diffusion of atoms into the porosity sites of the cryomilled powders [54].

**Figure 15:**
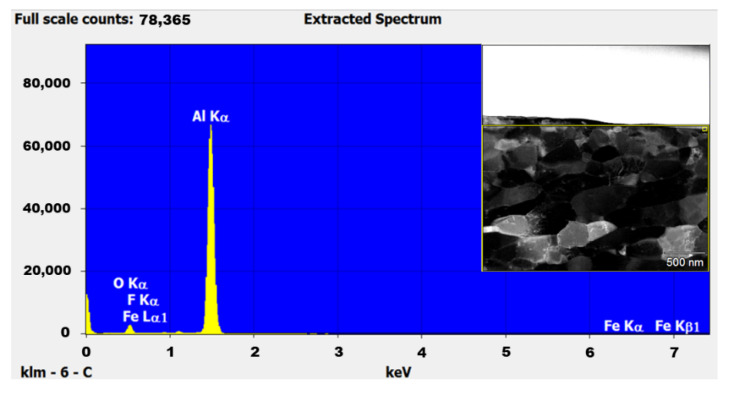
EDS plot for the unmilled SPS sample and the corresponding bright field image of the sample (inset image) as determined using TEM.

**Figure 16:**
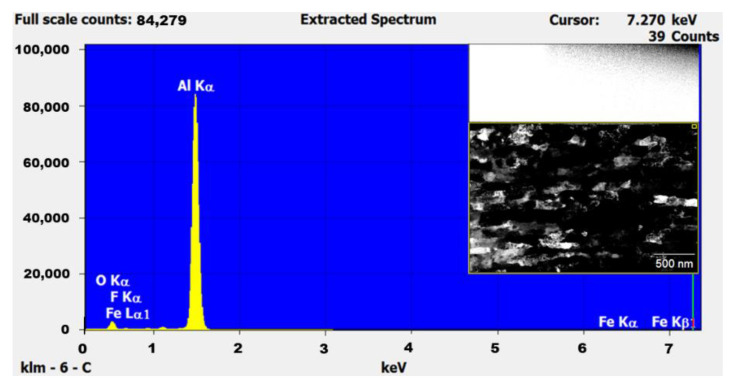
EDS plot for the 8 h cryomilled SPS sample and the corresponding bright field image of the sample (inset image) as determined using TEM.

EDS studies were also conducted using TEM on unmilled and cryomilled SPS samples to determine any phase changes or contamination during the SPS process. The EDS of the unmilled pure Al SPS sample showed the presence of around 96.58% aluminum and 2.71% oxygen, as shown in Figure 15. The EDS of the 8 h cryomilled pure Al SPS sample showed the presence of around 97.11% aluminum and 2.14% oxygen, as shown in Figure 16. The elemental values for both the samples are nearly the same, indicating a minimum effect of cryomilling and SPS on the elemental composition of the prepared bulk samples. The reduction in the oxide film is attributed to the high sintering temperature which is responsible for breaking down the oxide layers on the aluminum powder particle surface during the sintering process. Other minor elements (less than 1%) plotted in the EDS extracted spectrum are false positive peaks detected by the EDS.

#### Mechanical Properties of the Bulk SPS Consolidated Samples

Hall–Petch strengthening is the determining mechanism responsible for superior mechanical properties in NC materials. The reduction in crystallite size accommodates more grain boundaries, which eventually act as a barrier to the movement of dislocations in the grain structure of the NC materials. This large dislocation pile-up reduces the plastic flow of the material, thus increasing the strength and hardness of the material. The Hall–Petch relationship in terms of hardness is given by the following Equation (4).

*H_y_* = H_0_ + k*d*^−1/2^ (4)

Here, k and H_0_ are the empirical constants. The hardness (*H_y_*) of the material is inversely proportional to the square root of the crystallite size (*d*). Therefore, at lower values of crystallite sizes, the hardness will be very high. Hence, a little change in crystallite size below 50 nm causes a much larger change in the mechanical properties.

To determine the mechanical properties, Vickers microhardness tests were performed on the bulk SPS samples. Over 10 indentations were taken for each of the samples that were uniformly spread all over the polished surface. Hardness data has excluded the values that were significantly lower than the average. It is assumed that these indents were made on a pore zone. Table 4 shows the Vickers microhardness test results for the unmilled and the 8 h cryomilled SPS samples. The Vickers microhardness for the 8 h cryomilled pure Al SPS sample is 142 HV and for the unmilled pure Al SPS sample, it is 52 HV. Thus, the Vickers microhardness increases almost 3 times from the unmilled SPS sample to the 8 h cryomilled SPS sample. This drastic increase in the microhardness is due to plenty of fine grain accumulation on or near the grain boundaries that acts as a hindrance to crack propagation and dislocation movement, thus enhancing the hardness property of the cryomilled material [55]. The densification of the bulk sample during the SPS process is increased by severe plastic deformation, viscous flow, diffusion, and creep that eliminates pores and crevices, further increasing the hardness of the materials [35]. It is also observed that there was no hardness gradient in values in the radial direction of the polished SPS sample surface, indicating uniform temperatures across the component during the SPS process.

## 4. Conclusions

The current study demonstrated the manufacturing methodology for NC pure Al parts using a combination of cryomilling and SPS. The SPS parts produced exhibited NC grain size that provided superior properties to the bulk samples. This study provided a good understanding of the cryomilling process to reduce the crystallite size of the metal powders. It also helped to understand the annealing mechanisms involved in bulk component manufacturing, which could be incredibly useful for manufacturing other NC bulk alloys with superior mechanical properties. The following conclusions can be drawn from the current study:

1. Pure Al powders showed a 35% decrement in the crystallite size after 8 h of cryomilling determined using XRD. TEM studies also showed a decrease of about 72% in crystallite size. Thus, both XRD and TEM studies confirm the reduction in the crystallite size with increasing cryomilling time.
2. There was an increase in crystallite size with the increase in annealing temperatures due to grain coarsening, however, the crystallite sizes were still very well in the NC range. This ensured that the samples could withstand short heat treatment cycles without undergoing much change in the crystallite size.
3. Sintering temperature and pressure are crucial factors during the SPS process that ensures the uniform density and preservation of NC grains in the manufactured parts. The crystallite size increased by 55% after SPS for the 8 h cryomilled sample. The high sintering temperature helps in breaking down the oxide film on the powder particle surface, which reduces the oxide composition in the bulk sample.
4. There was a three-fold increase in the hardness for the 8 h cryomilled SPS sample, which is attributed to the combined effect of reduced crystallite size during the cryomilling process and densification of the SPS sample due to severe plastic deformation, diffusion, viscous flow, and creep. No hardness gradient was observed in the radial direction of the SPS samples, suggesting uniform heating during the SPS consolidation process.

## Figures and Tables

**Table 1:** Calculated crystallite sizes for different XRD peaks as determined from Scherrer’s equation.

Milling Time	L–XRD (nm)	L–TEM (nm)
Unmilled	43	67
2 h	35	27
6 h	31	26
8 h	28	19

**Table 2:** Crystallite size measurement using XRD for 8 h cryomilled pure Al powders using Scherrer’s equation.

Annealing Temperature	L_111_–XRD (nm)	L_200_–XRD (nm)	L_220_–XRD (nm)	L_311_–XRD (nm)	L_222_–XRD (nm)	Average L (nm)
300 °C	36	31	25	22	23	27
400 °C	39	34	26	23	24	29
500 °C	44	38	30	26	27	33
550 °C	46	40	31	27	27	34

**Table 3:** XRD crystallite size measurements for SPS samples as determined from Scherrer’s equation.

Milling Time	L_111_–XRD (nm)	L_200_–XRD (nm)	L_220_–XRD (nm)	L_311_–XRD (nm)	L_222_–XRD (nm)	Average L (nm)
Unmilled SPS	57	53	73	65	68	63
2 h SPS	59	57	64	56	62	60
8 h SPS	48	40	34	50	44	43

**Table 4:** Vickers microhardness results for unmilled and 8 h cryomilled SPS samples.

Sample	Vickers Microhardness (HV)
Unmilled Al SPS	52 ± 1
8 h cryomilled Al SPS	142 ± 12

## Data Availability

Not applicable.

## Abbreviations

EDS	Energy dispersive X-ray spectroscopy
FCC	Face centered cubic
FFT	Fast fourier transform
FWHM	Full width at half maximum
HEBM	High energy ball milling
HIP	Hot isostatic pressing
HRTEM	High resolution transmission electron microscopy
HV	Vickers microhardness
NC	Nanocrystalline
RPM	Rotations per minute
SAD	Selected area diffraction
SEM	Scanning electron microscopy
SPD	Severe plastic deformation
SPS	Spark plasma sintering/sintered
SS	Stainless steel
TD	Theoretical density
TEM	Transmission electron microscopy
